# Goltz syndrome (focal dermal hypoplasia) with unilateral ocular, cutaneous and skeletal features: case report

**DOI:** 10.1186/1471-2415-10-28

**Published:** 2010-11-19

**Authors:** Addis Tenkir, Samuel Teshome

**Affiliations:** 1 Department of Ophthalmology, College of Public Health and Medical Sciences, Jimma University, Jimma, Ethiopia; 2 Save the Children USA, Addis Ababa, Ethiopia

## Abstract

**Background:**

Goltz syndrome or focal dermal hypoplasia (FDH) is an uncommon multisystem disorder. Herein, we report a typical case of FDH with unilateral ocular, cutaneous and skeletal features.

**Case Presentation:**

a 4-year-old girl presented with microphthalmos and iris coloboma of the left eye, facial asymmetry, and a low-set protruding ear. Cutaneous changes included hypopigmented atrophic macules on the left side of the face, chest, abdomen and limbs. Characteristic lobster claw deformity of left hand and oligodactyly and syndactyly of left foot were present.

**Conclusions:**

FDH usually affects both sides of the body. This case represents the unusual unilateral manifestation of the syndrome.

## Background

Focal dermal hypoplasia (Goltz syndrome) is a rare genetic disorder characterized by distinctive skin abnormalities and a wide variety of defects that affect the eyes; teeth; and skeletal, urinary, gastrointestinal, cardiovascular, and central nervous systems. It is usually X-linked dominant with in utero lethality in males [1].

Ocular manifestations of focal dermal hypoplasia (FDH) are many and occur in more than 40% cases reported [2].

FDH generally involves both sides of the body. Only few cases of unilateral FDH are previously published [3-5]. Herein, we report a 4-year-old girl with typical features of Goltz syndrome affecting only the left side of the body.

## Case Presentation

A 4-year-old girl was referred to the ophthalmology department of Jimma University Specialized Hospital, Ethiopia for the evaluation of a 'small left eye' since birth.

The perinatal history of the child was uneventful. Family history was negative for similar problems. History of miscarriages was also denied. Her growth and developmental achievements were satisfactory.

**Figure 1:**
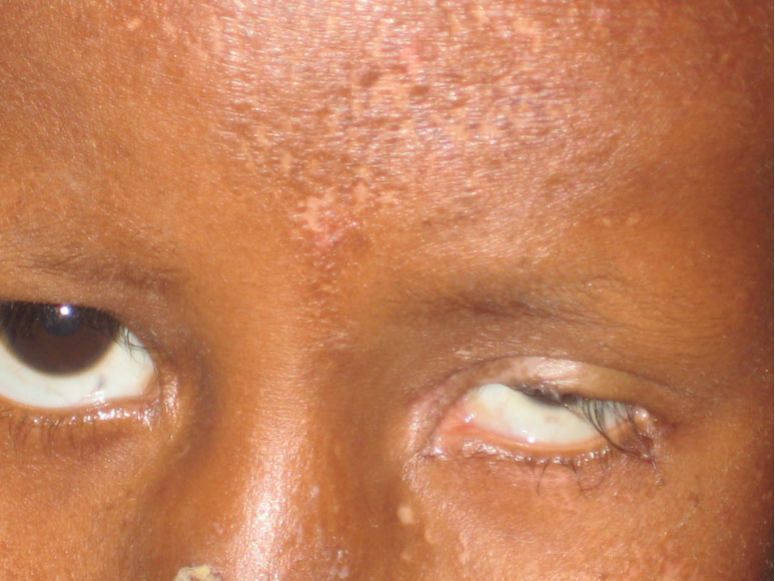
**Microphthalmia of the left eye**. Note also the hypopigmented atrophic macules on the forehead.

On examination, both eyes had simple infectious conjunctivitis. The right eye was within the normal limits apart from the conjunctival inflammation. The left eye was microophthalmic with typical inferior iris coloboma and cataractous lens (Figure 1).

**Figure 2:**
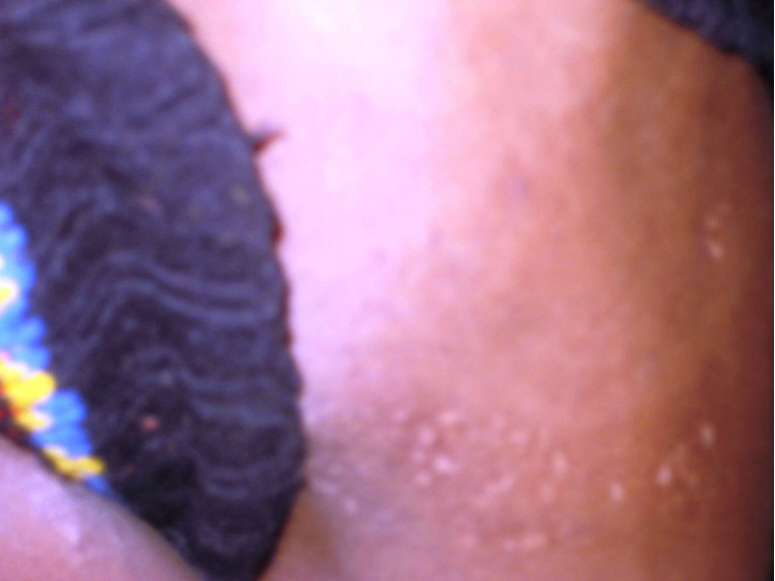
**Multiple hypopigmented, ill-defined atrophic macules on the left side of the trunk**.

Cutaneous examination showed multiple hypopigmented, slightly depressed and ill-defined macules over the left side of face (Figure 1). Similar skin lesions with reticular pattern were present on the side of chest, abdomen, buttock, and upper and lower limbs on the left side only (Figure 2).

**Figure 3:**
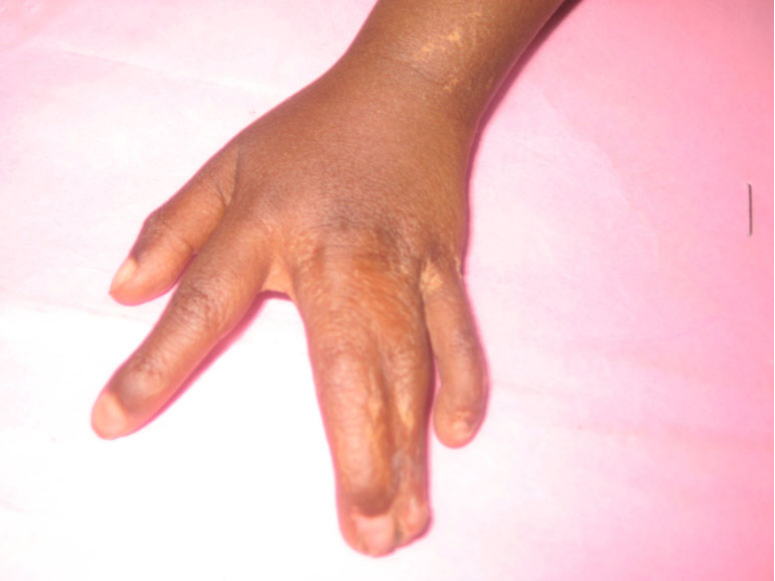
**Syndactyly with lobster claw deformity of the left hand**.

**Figure 4:**
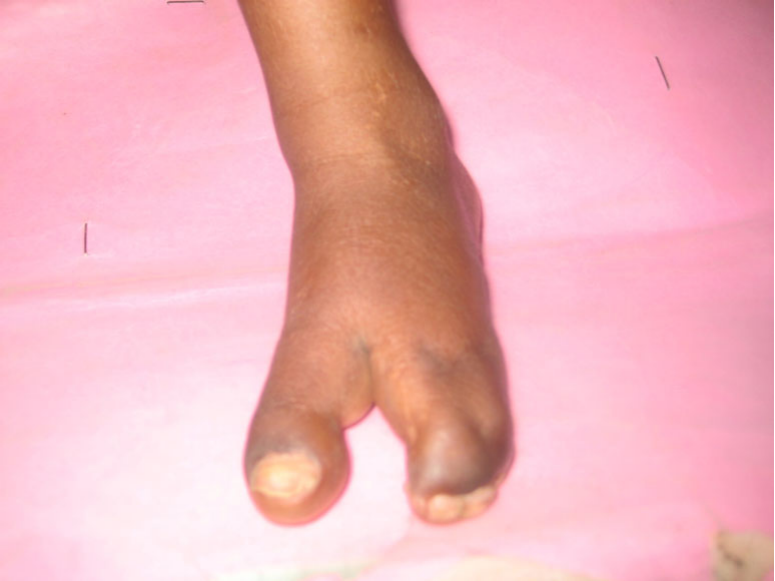
**Oligodactyly and syndactyly of the left foot**.

We noted complete syndactyly between the third and fourth fingers of the left hand, with typical lobster claw deformity (Figure 3). The left foot showed oligodactyly and syndactyly (Figure 4). The nails of the left hand and foot were dysplastic. The right hand and foot were within the normal limits.

There appeared to be facial asymmetry with left hemiatrophy, and low-set protruding left ear and pointed chin. The scalp hair was sparse; and the deciduous teeth were irregularly spaced (not shown in the pictures).

The simple bacterial conjunctivitis was treated with topical chloramphenicol eye drops. We arranged appointment for regular follow up.

## Discussion

Goltz syndrome is an uncommon multisystem disorder. Abnormalities are seen in multiple organ systems including the eyes [1].

Ophthalmic manifestations of FDH occur in 40% of cases. Ocular colobomas, strabismus and microphthalmia seem to be the most frequent manifestations of the syndrome. Other findings reported include anophthalmia, hypertelorism, ectropion, ptosis, nasolacrimal duct obstruction, lid margin or conjunctival papillomas, corneal clouding, blue sclera, aniridia, heterochromia, irregularity of the pupils, cloudiness of the vitreous, and optic nerve hypoplasia [2]. Multiple discrete vascularized peripheral subepithelial corneal opacifications [6], retinal neovascularization with recurrent vitreous hemorrhage [7], and anterior persistent hyperplastic primary vitreous [8] were also associated with Goltz syndrome.

FDH generally involves both sides of the body. Only few cases of unilateral FDH are previously published [3-5]. These include a 16-month-old girl with numerous malformations and cutaneous lesions on the right side of the body [3], a 7-year-old girl with a myriad of right side abnormalities on her face, hand and foot [4], and an 8-year-old girl with asymmetric body halves [5]. Our patient had a milder form of the disease. Lyonization and mosaicism are suggested as possible explanations for variability in severity of expression [9].

The differential diagnosis includes incontinentia pigmenti and microphthalmia, dermal aplasia and sclerocornea (MIDAS) syndrome. The clinical history of incontinentia pigmenti includes cutaneous vesiculation and verrucous lesions with hyperpigmentation, which differ from the linear atrophic areas of FDH. A higher proportion of patients with incontinentia pigmenti have convulsions and neurological deficits than those with FDH. MIDAS syndrome presents with microphthalmia, dermal hypoplasia and aplasia, which are limited to the upper half of the body, mainly the face and neck [8,10].

Treatment of Goltz syndrome is largely supportive. As it is a multisystem disease, a multidisciplinary approach, including reconstructive surgery, is required for effective management of these patients.

Dunlop and colleagues (1999) treated a girl who developed recurrent vitreous hemorrhage associated with Goltz syndrome by indirect retinal photocoagulation to the areas of non-perfusion with subsequent new vessel regression within weeks [7].

In a report of a case of FDH by Marcus and colleagues (1990), exenteration was required for an unsightly and chronically infected anophthalmic socket after previous attempts at surgically reconstructing the socket for the fitting of a prosthesis had failed [2].

## Conclusion

In conclusion, our patient manifests the typical features of Goltz syndrome affecting only the left side of the body.

## Consent

Written informed consent was obtained from the father of the patient for publication of this case report and the accompanying pictures. A copy of the written consent is available for review by the Editor-in-Chief of this journal.

## Competing interests

The authors declare that they have no competing interests.

## Authors' contributions

AT drafted the manuscript. Both AT and ST reviewed, read and approved the final manuscript.

## Authors' information

**Addis Tenkir, MD**. Assistant Professor, Department of Ophthalmology, Collage of Public Health and Medical Sciences, Jimma University, Jimma, Ethiopia.

**Samuel Teshome, MD, DLSHTM**. Save the Children USA, Addis Ababa, Ethiopia.

## Pre-publication history

The pre-publication history for this paper can be accessed here:

http://www.biomedcentral.com/1471-2415/10/28/prepub
